# The outstanding scientist, R.A. Fisher: his views on eugenics and race

**DOI:** 10.1038/s41437-020-00394-6

**Published:** 2021-01-15

**Authors:** Walter Bodmer, R. A. Bailey, Brian Charlesworth, Adam Eyre-Walker, Vernon Farewell, Andrew Mead, Stephen Senn

**Affiliations:** 1 Oxford University, Weatherall Institute of Molecular Medicine, John Radcliffe Hospital Oxford, Oxford, OX3 9DS UK; 2 School of Mathematics and Statistics, University of St Andrews, Scotland, KY16 9SS UK; 3 Institute of Evolutionary Biology, School of Biological Sciences, University of Edinburgh, Charlotte Auerbach Road, Edinburgh, EH9 3FL UK; 4 School of Life Sciences, University of Sussex, Brighton, BN1 9QG UK; 5 Cambridge, UK; 6 Computational and Analytical Sciences, Rothamsted Research, Harpenden, Hertfordshire, AL5 2JQ UK; 7 Edinburgh, UK

**Keywords:** Genetics, Evolution

## Introduction

**Fig. 1:**
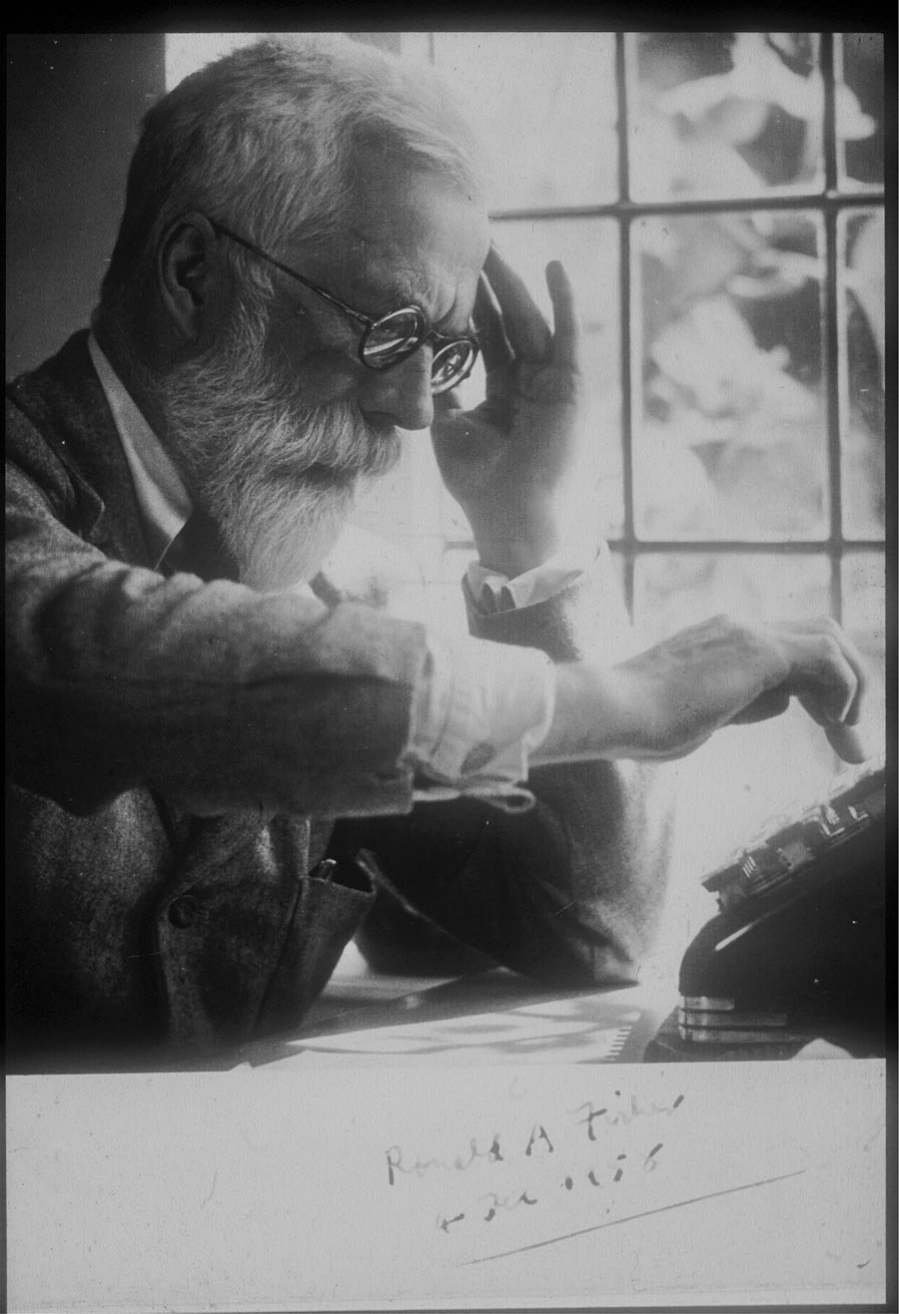
R.A. Fisher at his calculator in 1958 (courtesy of the Fisher Memorial Trust).

R.A. Fisher was one of the greatest scientists of the 20th century (Fig. 1). He was a man of extraordinary ability and originality whose scientific contributions ranged over a very wide area of science, from biology through statistics to ideas on continental drift, and whose work has had a huge positive impact on human welfare. Not surprisingly, some of his large volume of work is not widely used or accepted at the current time, but his scientific brilliance has never been challenged. He was from an early age a supporter of certain eugenic ideas, and it is for this reason that he has been accused of being a racist and an advocate of forced sterilisation (Evans 2020). His promotion of eugenics has recently caused various organisations to remove his name from awards and dedications of buildings (Tarran 2020; Rothamsted Research 2020; Society for the Study of Evolution 2020; Gonville and Caius College 2020). A primary aim of this paper is to conduct a careful analysis of his own writings in these areas. Our purpose is neither to defend nor attack Fisher’s work in eugenics and views on race, but to present a careful account of their substance and nature.

## Fisher’s scientific achievements

### Contributions to statistics

Fisher has been described as “the founder of modern statistics” (Rao 1992). His work did much to enlarge, and then set the boundaries of, the subject of statistics and to establish it as a scientific discipline in its own right. Much of his inspiration in developing statistics came from practical applications in a variety of scientific areas, notably in agriculture, where he developed techniques arising out of his work at the Rothamsted agricultural research establishment (then Rothamsted Experimental Station, now Rothamsted Research), and later in mouse and human genetics.

Fisher was the first to point out the fundamental distinction between a *statistic* and a *parameter* and pioneered the statistical concept of *likelihood* and related ideas central to any theory of estimation. He extended the use of Student’s *t* test, developed the theory of significance testing, provided the correct interpretation for the use of Pearson’s chi-square goodness-of-fit test and developed an exact test, that now bears his name, for the 2 × 2 contingency table. Fisher coined the term ‘variance’ in his famous 1918 paper on Mendelian genetics (Fisher 1918a, see next section) and the concept of the analysis of variance, often now referred to as ‘anova’. His first book, *Statistical Methods for Research Workers* (Fisher 1925), which appeared in 1925, eventually went into 14 editions and was read by and influenced research workers in many subjects.

He was the first to propose a systematic, scientific approach to the design and analysis of experiments, pioneered in his classic and still highly readable book *The Design of Experiments*, first published in 1935 (Fisher 1935a). He promoted and formalised understanding of close local control (often referred to as blocking) and replication, as ways of increasing the reliability of inferences on the effects of treatments. He pointed out that more information could be gained from an experiment using all combinations of levels of two or more treatment factors (such as sowing date and amount of manure) than from consecutive experiments which each investigated one factor at a time. He emphasised the importance of distinguishing between three sources of variation: experimental treatment, other observed (designed/planned) sources of variation (such as different fields) and unobserved sources of variation. In order to deal with the latter, Fisher also championed the need for randomisation to obtain valid and unbiased estimates of effects (for example, of applications of fertilisers in plant breeding experiments or clinical trials of drug treatments for testing their efficacy) and also valid estimates of their reliability. Fisher particularly emphasised the importance of having statisticians embedded in agricultural, medical and other research institutes, an approach that helped to widen the successful application of his many innovative statistical developments.

He had extraordinary geometrical insight and made major contributions in probability applied to multi-dimensional problems, the subject now called multivariate analysis. An example of this is his work on distributions on a sphere, which proved useful to other investigators studying directions of magnetism in the earth’s rocks in order to understand continental drift. Fisher also developed the mathematics of several key probability distributions and produced, with Yates, a pioneering and widely used collection of statistical tables (Fisher and Yates 1938).

The widespread applications of Fisher’s statistical developments have undoubtedly contributed to the saving of many millions of lives and to improvements in the quality of life. Anyone who has done even a most elementary course in statistics will have come across many of the concepts and tests that Fisher pioneered. Unfortunately, the only reason many say they have heard of Fisher is because of his exact test for the analysis of 2 × 2 tables!

### Contributions to genetics

Fisher made pathbreaking contributions to genetics and evolutionary biology. From an early age, he was fascinated by Darwin’s work on evolution. As an undergraduate in the 1910s, he became acquainted with the new science of genetics and quickly realised that the particulate nature of inheritance revealed by the crossing experiments of Gregor Mendel and his successors removed the problem that had bedevilled Darwin—the loss of variability required for the effectiveness of natural selection under the “blending inheritance” mechanism that was assumed by Darwin.

Fisher’s first major contribution to genetics was his revolutionary 1918 paper entitled “The correlation between relatives on the supposition of Mendelian inheritance” (Fisher 1918a, see also Fisher 1918b). He showed how Mendelian genetics could explain the patterns of correlations among relatives in quantitatively varying traits like height, on the hypothesis that many different genetic factors contribute to such quantitative variation, together with non-genetic factors. Fisher introduced the mathematical machinery that allows the decomposition of variation into different causal components. This has formed the underpinning of research into the genetics of complex traits for the last 100 years, with important applications to animal and plant breeding, and the genetic analysis of many human diseases and disorders.

Genes are most simply defined as segments of our DNA sequence that control particular biochemical functions. Different individuals can have different versions of any given gene and it is the extent to which this occurs that determines the wide range of genetic variation in human populations. When two or more versions of a given gene occur with frequencies of about 1% or more in a population, they are called genetic polymorphisms, from the Greek for many (poly) and forms (morph). The genetic relationships between populations are defined by their patterns of differences in polymorphism frequencies, and the study of these frequencies is a major focus of the field of population and evolutionary genetics that was pioneered by Fisher and two other notable contemporary geneticists, J. B. S. Haldane and Sewall Wright (Provine 1971).

To understand the population processes responsible for maintaining variation in quantitative traits, Fisher pioneered mathematical models of how both natural selection and random fluctuations (due to the sampling effects of finite population size) affect the frequencies of genetic polymorphisms in populations (Fisher 1922, 1930b). These papers form the conceptual framework for modern theoretical work on the genetics of populations, providing the basis for our understanding of how evolution works. One of Fisher’s important discoveries was that selection can preserve variation in the population rather than eliminating it (Fisher 1922); this is important for the understanding of certain human disorders such as sickle cell disease (for a general background on human genetics, see Bodmer and Cavalli-Sforza 1976).

Fisher’s seminal early work synthesising genetics and evolution was summed up in his 1930 book *The Genetical Theory of Natural Selection* (Fisher 1930a). This is full of brilliant insights into the nature of the evolutionary process, including ideas on the evolutionary significance of sexual reproduction and genetic recombination, the evolutionary reason for the 1:1 sex ratio, and the evolution of ageing, as well as the formalisation of the theory of natural selection in his famous “Fundamental Theorem of Natural Selection”. It is widely regarded as the most original book on evolution after Darwin’s *Origin of Species* (Darwin 1859). This work of Fisher’s, together with that of J.B.S. Haldane and Sewall Wright, rescued Darwin’s theory of evolution by natural selection from the neglect into which it had fallen (see Provine 1971).

During the 1930s, Fisher turned his attention to human genetics and developed influential statistical methods for analysing linkage between human genes. Fisher was the first to promote the study of the human blood groups in the UK as a systematic approach to human genetics at the biochemical level and through this pioneered their use for studying human population genetics. An outcome of this work was Fisher’s masterly analysis of the genetics of the Rhesus blood group system (Fisher 1947), which brought order to a chaos of data. Incompatibility between the Rhesus make-up of the mother and foetus is a major cause of haemolytic disease of the newborn; understanding the genetics of Rhesus has enabled this disease to be largely prevented (Baskett 2019).

It should be noted that the development of the science of population genetics by Fisher, Haldane, Wright and their contemporaries led to the understanding that different populations of the same species differ with respect to the frequencies of genetic polymorphisms at many different places in the genome, rather than each population being a homogeneous entity. This completely undermined any concept of racial purity, of the type advocated by the Nazis and white supremacists, a point vigorously made in arguments against racism from the 1930s to the 1950s by scientists including Theodosius Dobzhansky, Gunnar Dahlberg, J.B.S. Haldane, Lancelot Hogben, Julian Huxley, H.J. Muller and Ashley Montague (Neel and Schull 1954, p 256; Kevles 1985).

## Fisher’s support for eugenics, and related issues

### Fisher’s introduction to genetics and eugenics in Cambridge

The year 1909, when Fisher began to study mathematics in Cambridge, was the 50th anniversary of the publication of Darwin’s *Origin of Species* (Darwin 1859) and the year that William Bateson’s book *Mendel’s Principles of Heredity* (Bateson 1909) was first published. Fisher became interested in biology while a teenager and was an avid reader of Charles Darwin’s writings, having received his complete works as a school leaving prize (Box 1978). Fisher chose to study mathematics rather than biology because he thought that for a future biologist “a mathematical technique with biological interests is a rather firmer ground than a biological technique with mathematical interests” (Crow 1990). In Cambridge, at Gonville and Caius College, he quickly absorbed the new subject of Mendelian genetics, and at the same time became interested in Galton’s ideas on what in 1883 he had called eugenics, by which he meant the science of improving the “human stock” (see quotation below). Both of these subjects were represented amongst the Fellows at Gonville and Caius at that time.

Galton introduced the word eugenics as follows: “We greatly want a brief word to express the science of improving stock, which is by no means confined to questions of judicious mating, but which, especially in the case of man, takes cognisance of all influences that tend in however remote a degree to give more suitable races or strains of blood a better chance of prevailing speedily over the less suitable than they otherwise would have had. The word *eugenics* would sufficiently express the idea;” (Galton 1883, pp 24–25). The aim of eugenics was therefore to be the “science of improving [the human] stock”.

Galton’s approach to inheritance was to study correlations between parents and offspring for quantitative traits, not only height and weight but also measures of a person’s intelligence, which later became the IQ test (Cavalli-Sforza and Bodmer 1971, pp 514–517; Provine 1971). These correlations constituted the biometric approach to studying the inheritance of the traits being measured, which became a key component of later research into the genetics of quantitative traits. An early challenge for the application of eugenics was then to encourage marriages between individuals of presumed high intellectual calibre, something that nowadays would not be considered acceptable as an official policy in most societies. It is important to emphasise that this original meaning of eugenics had nothing to do with its later odious connotation of Nazi racist policies, most viciously directed against the European Jews.

Fisher became enthusiastic about the potential for eugenics based on Mendelian inheritance and so was instrumental in founding The Cambridge University Eugenics Society (Box 1978, pp 26–27). During the first term of his third year as an undergraduate in Cambridge he gave a remarkable talk to the society entitled “Mendelism and Biometry”, which foreshadowed many of his later outstanding contributions to genetics (Bennett 1983, pp 51–63). He first gave an outline of Mendelian genetics and how it could be related to the biometrical study of inheritance and used to explain the correlations between parents and offspring observed by Galton. This was the main subject of what is perhaps Fisher’s most famous genetical paper (Fisher 1918a), written in 1916 but not published until 1918, which contains no mention of eugenics. His aim in that paper was to resolve the apparent conflict between the Mendelian and biometrical approaches to the study of inheritance.

### Fisher’s support for a eugenic approach to correcting the inverse relationship between fertility and its achievement through family allowances

In his “Mendelism and Biometry” talk, Fisher said “The interest of the biometrical work for eugenists lies in the fact that Francis Galton employed this method, the only one then open to him, to show that human characters are as strongly inherited as those of animals, and mental characters as much as physical… in *Hereditary Genius*, Galton shows how strongly such talents are inherited; and it is of the utmost importance to select such men from whatever class they may be born in, to enable them to rise in the world, to encourage them to marry women of their own intellectual class, and above all to see that their birth-rate is higher than that of the general population….but at present, there is no doubt that the birth-rate of the most valuable classes is considerably lower than that of the population in general,… the mental power should be closely examined in a uniform environment, for instance of the elementary schools, and that special facilities should be given to children of marked ability” (Bennett 1983, pp 57–58).

It was Fisher’s concern, as quoted above, at the inverse relationship between the birth-rates of the ‘most valuable classes’ and the ‘lower classes’, in which he included, for example, skilled labourers, that dominated Fisher’s involvement in eugenics and the Eugenics Society for the next 20–25 years. His concept of class, as was not uncommon at that time, had nothing to do with modern racial concerns. The “upper classes” were characterised by Fisher by their intellectual capacity and education rather than money or heritage, as discussed in the next paragraph. This, of course, ignores the fact that access to a good education in Britain was, and still is, although to a much lesser extent, heavily biased towards the well-off, and that economic success is greatly assisted by parental wealth. Ironically, in view of Fisher’s conservative political views, a policy of encouraging equality of fertility between the “upper classes” and “lower classes” could only be effective under an equitable economic system, as was pointed out in “The Geneticists’ Manifesto” of the 1939 International Congress of Genetics (to which Fisher was not a signatory) (Crew et al. 1939). This document was signed by 23 leading geneticists, including some with strongly left-wing political views like J.B.S. Haldane, H.J. Muller and Lancelot Hogben. It started with the question “How could the world’s population be improved most effectively genetically?”. They went on to say that “the raising of the level of the average of the population nearly to that of the highest now existing in isolated individuals …. would, as far as purely genetic considerations are concerned, be possible within a comparatively small number of generations”. This goes far beyond the proposal of Fisher’s described below, and shows that eugenic ideas were widely held across the political spectrum at the time (see Paul 1984 for further discussion).

These presumed “dysgenic” effects of the inverse relationship between fertility and achievement were the basis for Fisher’s explanation for the decline of civilisations, such as the Egyptians and Babylonians, expounded at some length in the last five chapters of *The Genetical Theory of Natural Selection* (Fisher 1930a), and this is what is most relevant to his eugenic interests. The discussion starts with emphasising that humans are subject to the same laws of inheritance as all other animals and that these laws apply equally to “the mental and moral qualities” as to the more obvious physical attributes (p 186). He then discusses the evidence for the inheritance of human fertility and how important variation in fertility is for the action of natural selection, especially in human populations (pp 194–199). He presents evidence for social class as “defined by the aggregate of persons or families, inter-marriage with whom will encounter no social obstacles” being strongly correlated with fertility (p 211), and that social class is significantly determined by genetic factors correlated with “brain-workers” not particularly “titled families” (pp 211–212). Based on his view that the differences between social classes are largely due to differences in inherited abilities, he argues that the inverse relationship between class and fertility in any given civilisation will lead to a gradual and inevitable decline in the proportion of people who have the abilities that define the higher classes, and are required for outstanding leadership. Fisher then argues that this decline in leadership qualities could be the explanation for the eventual decline of a civilisation (pp 228–242).

All of these arguments relate to variation within, and not between populations, and so have nothing directly to do with racial differences, however races are defined. There is an indirect relation, though. Fisher argued that pre-civilisation societies were more conducive to maintaining genetic quality with respect to the leadership traits in question (Fisher 1930a, pp 245–252, 254). A logical consequence of his arguments is thus that such societies are more likely to have high overall quality than civilised ones, which might be seen as the inverse of what Western racists would have us believe, although Fisher himself did not mention this point. His answer, as a eugenist, to preventing the decline in leadership qualities was to promote family allowances in a way that encouraged the ‘higher’ classes to have more children: “…if family allowances were paid at a rate which on the average allowed an equal standard of living between parents and non-parents it follows that the standard of living of the competent will rise, and that of the incompetent will fall, with increasing size of family;.” This is how Fisher put it in a letter to his strong supporter and effective mentor, Leonard Darwin, Charles Darwin’s second youngest son (Bennett 1983, p 133).

That Fisher was not class conscious in a conventional sense is made clear in a 1935 letter advocating “The fashion of attaching prestige not to ancient pedigree or wealth, but to biological fitness and readiness for parenthood” (Fisher 1935b, see also Fisher 1930a, pp 210–211). He did not think education should be constrained by class, and was himself supported by scholarships at Harrow School and Cambridge University (Box 1978, pp 14–18). As he put it in a 1930 letter to E.B. Wilson, “The more thoroughly we carry out the democratic programme of giving equal opportunities to talent wherever it is found, the more thoroughly we insure that genetic class differences of eugenic value shall be built up” (Bennett 1983, p 272). Another example of Fisher’s approach to promoting eugenic practice was his argument against “joint assessment of husband’s and wife’s income for income tax purposes” as it “is a definite penalisation of marriage, especially affecting the professional classes in their early years” (Correspondence, digital.library.adelaide.edu.au 1935-00-00). This is because of the fact that if, for example, the wife’s is the lower income this will be taxed at the higher rate corresponding to the husband’s income if the two incomes are combined. This change in taxation was enacted in Britain in 1989, but obviously without reference to its eugenic potential!

Fisher emphasised the difficulty of finding good data that correlate social class with fertility and campaigned for the 1931 census to include questions that would improve the quality of such fertility data. Subsequent analyses by Cavalli-Sforza and Bodmer (1971), using data from C.J. Bajema (1963), no longer supported the presumed simple inverse relation between IQ and fertility and the assumption that this would lead to a decline in IQ, as implied by Fisher.

### Fisher’s active support for the Eugenics Society and for its policy of voluntary sterilisation for the “feeble minded”

For many years Fisher promoted his eugenic ideas through his activity as a member of the Eugenics Society and its council. The Eugenics Society was founded 1907 as the Eugenics Education Society, largely on the suggestion and encouragement of Sybil Gotto. (See Mazumdar 1992 for a history of the society, based on its own documents.) Francis Galton was its first President, and Leonard Darwin became President in 1911. It became the Eugenics Society in 1924. One of the issues strongly supported by Fisher and the Eugenics Society was the aim to prevent an increase in inherited “feeble mindedness” or “grave transmissible defects” by offering voluntary sterilisation. This is often referred to as negative eugenics. Legislation for voluntary sterilisation was strongly supported (Brock 1934), but never enacted in Britain. Fisher made clear his support for the sterilisation being strictly voluntary in a draft letter to the Dean of St Paul’s, who had referred to sterilisation as mutilating, which Fisher countered by saying it was less mutilating than “drawing a tooth”. He then continued, “The horrible associations of the word mutilation are inappropriate because the patient voluntarily undergoes the operation and we do not urge the legalisation of eugenical sterilisation save with the consent of the patient.” (Bennett 1983, pp 79–80).

R. C. Punnett, Bateson’s protégé and successor in Cambridge, as the Professor of Genetics and a Fellow of Trinity College, argued that, assuming recessive inheritance, sterilisation to remove the contribution of the homozygous individuals, who carry two copies of the abnormally functioning deleterious version of the relevant gene, would have minimal effects on the reduction of their incidence (Punnett 1917). Taking advice from his friend, the famous mathematician GH Hardy (of Hardy–Weinberg law fame), who was also a Fellow of Trinity College, he produced a table showing that, for a recessive allele with a population frequency of 1%, it would take 22 generations of completely preventing the recessive trait individuals from reproducing to reduce their frequency to 1/1000 and another nearly 1000 generations to bring the frequency down to 1/1,000,000. Punnett then said, based on similar calculations, that “If the proportion of feeble-minded in the United States is 3 per 1000 today it would require something over 250 generations, or about 8000 years before the proportion was reduced to 1 in 100,000, and nearly four times as much before the feebleminded were as few as I in a million”.

These very slow rates of reduction of the frequencies of the “feeble-minded”, based on the model of their being due to a single recessive allele are because the rare recessive homozygotes are overwhelmingly produced by matings between heterozygotes, who carry one deleterious and one normal copy of the relevant gene, so that preventing homozygotes from having children has a minimal effect on the frequency of heterozygotes. Fisher countered Punnett’s argument in his 1924 article on “The elimination of mental defect” by first calculating that, using Punnett’s frequency of 1% for the frequency of deleterious homozygous recessives, corresponding by the Hardy–Weinberg law, to an allele frequency of (1/100)^1/2^ = 10%, one generation of preventing the affected recessives from producing offspring would reduce their incidence by 17%, for example from 100/10,000 to 83/10,000 (Fisher 1924; see also Cavalli-Sforza and Bodmer (1971), p 181) for a formula for these calculations). This is a figure wrongly quoted by the historian Sir Richard Evans as being Fisher’s estimate of the proportion of British “defectives” (Evans 2020). Fisher then said “If our starting point had been 30 instead of 100, still a single generation of selection would lighten the burden by over 11%”, using here Punnett’s frequency [of affected individuals] of 3/1000, rather than the 1% of Fisher’s initial calculations.

Fisher next argued against Punnett’s assumptions, “(1) That feeblemindedness may be equated to a mendelian recessive, (2) that the population chooses its mates *at random.”* This latter assumption is implicit in the use of the Hardy–Weinberg law for the calculations. Fisher first supported his argument against Punnett’s assumptions by making the case that while “There is a considerable body of pedigree evidence indicating the existence of a single mendelian factor which, in its recessive phase, is capable alone of producing feebleness of mind. … Consequently, while we may speak of feeblemindedness as due to a Mendelian recessive, no responsible authority would claim all the feebleminded cases are genetically alike.” He finished this argument with the statement “Consequently in the case of so variable a characteristic as feebleness of mind, it would be extremely rash to assume that only one main factor is present and entirely contrary to the evidence to ignore the contributions of less important factors”. Fisher next argued that assortative mating among the feebleminded (so that the frequencies of matings between them would be greater than with random mating), would substantially increase the rate of reduction in the incidence of the feebleminded by sterilisation. This is because all offspring from matings between affected individuals are themselves affected, whereas most offspring of matings between affected and unaffected individuals are unaffected. This argument was used by the Eugenics Society to support their case for the voluntary sterilisation legislation. It is now abundantly clear that Punnett used too high a frequency for a trait caused by a single deleterious recessive allele and was wrong in assuming this as the explanation for “feeble mindedness”, while subsequent work on the genetics and biology of mental disabilities has, as Fisher proposed, revealed the complex nature of their causalities (Penrose 1949).

Following Parliament’s rejection of the bill promoted by the Eugenics Society for the legalisation of voluntary sterilisation, the British Government set up a committee in 1932, chaired by Laurence Brock, to investigate the issue further (Brock 1934). Fisher was a member of the committee representing the Eugenics Society, as was Ruth Darwin, a granddaughter of Charles Darwin. Fisher, on behalf of the committee, carried out an extensive analysis of the data on the patterns of incidence and inheritance of “Mental Defectives” provided by “returns from the local authorities” (Fisher 1934). The “Brock Committee” came out clearly against compulsory sterilisation but unanimously supported the legalisation of voluntary sterilisation, subject to certain safeguards, for “a person who is mentally defective or has suffered from severe mental disorder; a person who suffers from, or is believed to be a carrier of, a grave disability which has been shown to be transmissible; and a person who is believed to be likely to transmit mental disorder or defect.” The safeguards included, firstly, that the consideration of whether an individual’s disability justifies making the case for voluntary sterilisation be made by at least two medical practitioners, one of whom could be the ‘family’ doctor and, secondly, “If the practitioner is not satisfied that the patient is competent to give a reasonable consent, the full consent and understanding of the parent or guardian should be obtained”. The recommendations of the Brock report were, however, never enacted.

Elective abortion with prenatal testing and pre-implantation diagnosis were not available until the late 1950s, after the development of amniocentesis (see e.g. Bodmer and Cavalli-Sforza 1976), but have now become readily available as choices, in addition to sterilisation, for parents who wish to avoid having children who are severely disabled. The fundamental difference is that now these choices are made for the sake of the parents and their offspring, not for the overall benefits to society. It is worth noting that it was Fisher, in 1935, who first made the suggestion, using the dominantly inherited Huntington disease as an example, that linked markers might be used for predicting the presence or absence of the abnormal gene in the offspring of a known carrier (Fisher 1935c). This has now become widely possible through the huge technical developments of DNA sequencing, which enables the ‘positional cloning’ of a gene whose abnormality causes an inherited disease, based only on the knowledge of where the gene lies in the human genome.

Fisher did not pursue the campaign for legalisation of voluntary sterilisation and his other eugenic interests after 1934/5, apart from one further paper in 1943 on family allowances at a time when there was considerable concern at the low overall birth-rate in the UK (Fisher 1943). He became increasingly disillusioned with the activities of the Eugenics Society because of its apparently increasing lack of scientific direction. This was reflected in Fisher’s earlier criticism of Lidbetter’s analysis of his pedigree study of paupers in East London, pointing out that Lidbetter had no control group and no collection of data that would enable environment to be distinguished from heredity as a cause of poverty (Mazumdar 1992, pp 93–93, 97–98). Fisher also became influenced by Penrose’s classical studies on mental disabilities (see Kevles 1985, pp 166, 344; Penrose 1949). At that time, in the early 1930’s, he was also developing his interest in blood groups and Mendelian inherited human traits and their linkage relationships. In a letter to P.F. Fyson in 1938, Fisher wrote, “I do not see that much can be done with the Eugenics Society, as its present directors of policy are strongly entrenched and appear almost impervious to scientific advice” (Bennett 1983, p 206). In 1941, in a formal letter to the Eugenics Society, he wrote, “As you know, I have for some years taken no part in the work of the Eugenics Society, although from time to time my name has been put on the council. I am afraid now that I must dissociate myself more distinctly from the society than has hitherto seemed necessary, and write to let you know that I do not wish my name to appear as a member of the council, or in any connection other than the Consultative Council which, I understand has no responsibility for the society’s actions.” (letter to Mrs. Collier, June 30th, 1941, Wellcome Library).

### Fisher’s interaction with the Nazi-supporting medical geneticist, Verschuer

There are two further incidents related to racism, on the basis of which Fisher has been criticised. The first concerns his interaction with the German human geneticist Otmar Freiherr von Verschuer, who has been strongly condemned because of his involvement with Nazi racial policies, most horrifyingly directed against Jews (Weiss 2010). Fisher first corresponded with Verschuer in early 1938 concerning a visit to London, which Verschuer made in June,1939, remarkably less than 3 months before the start of World War II (letters to Verschuer 1938-03-10, 1939-05-27). Fisher’s next contact was in 1947 when Verschuer approached him, as he did many others (Weiss 2010), for a reference in support of his post-war campaign to re-establish his position as a human geneticist by being made a professor in the University of Frankfurt. In reply, Fisher wrote “Please let me know if I can do anything to help you.”…“something in the nature of a certificate of character, i.e. an assertion that I know you to be a genuine man of science of reputation and merit, and believe you not to be subversive to the peace of Europe.” (letter to Verschuer 1947-08-070). Fisher’s reference letter to Wezler, the Dean of the Medical Faculty, said, “As he has been attacked for sympathy towards the Nazi movement, I may say that his reputation stood exceedingly high among human geneticists before we had heard of Adolph [*sic*] Hitler. It was, I think, his misfortune rather than his fault that racial theory was a part of the Nazi ideology, and that it was therefore of some propaganda importance to the Nazi movement to show that the Party supported work of unquestioned value such as that which von Verschuer was doing. In spite of their prejudices I have no doubt also that the Party sincerely wished to benefit the German racial stock, especially by the elimination of manifest defectives, such as those deficient mentally, and I do not doubt that von Verschuer gave, as I should have done, his support to such a movement. In other respects, however, I imagine his influence was consistently on the side of scientific sanity in the drafting and administration of laws intended to this end.” (Weiss 2010, p 745).

These statements have been interpreted by some (e.g. Evans 2020) as suggesting that Fisher referred to elimination in the sense of killing or at least compulsory sterilisation or institutionalisation, and so was a Nazi sympathiser. This is, however, in obvious disagreement with his very clearly stated views that sterilisation should be voluntary and with his support for the Brock report.

Other referees for Verschuer, for example, the renowned geneticist Hermann Muller, although not recommending Verschuer due to his Nazi associations, referred to his respect for Verschuer’s genetic work, including his twin studies (Weiss 2010, pp 743–744), while even the refugee geneticist, Richard Goldschmidt, who had been forced to leave his position in Germany because he was Jewish, supported Verschuer as “a fine and sympathetic person” and “an exceptional scholar in his field and one of the most knowledgeable medical geneticists.” (Weiss 2010, p 745; Goldschmidt 1947). In a letter to Verschuer of 3 February 1948, Fisher wrote “It does not seem to be at all easy to arrange a visit to this country. There has evidently been a good deal of denigration, which I do not believe has any substantial basis.” (letter to Verschuer 1948-02-03).

While there were undoubtedly some people in the later 1940s who knew that Mengele, the Auschwitz doctor called “The Angel of Death” for his involvement in horrific human experiments, was a student of Verschuer and collaborated with him during the war, this was not generally widely known at that time and it seems quite possible that Fisher, perhaps naively and not wanting to believe the worst, gave Verschuer the benefit of doubt and supported him simply as a fellow human geneticist, as did Goldschmidt. Fisher’s last contact with Verschuer was in the late 1950’s when he was seeking data on smoking patterns in identical, monozygous, versus non-identical dizygous, twins (letter to Verschuer 1958-03-14). It seems strange, however, that Fisher apparently ignored, or was unaware of, the well-documented involvement of prominent German human geneticists in Nazi policies during the 1930s, including the notorious Eugen Fischer, Verschuer’s mentor and predecessor at the Kaiser Wilhelm Institute for Anthropology, Human Genetics and Racial Hygiene [eugenics] (Glass 1981; Weiss 2010).

### Fisher’s response to the UNESCO statement on “The Race Concept”

The second incident concerning Fisher’s alleged racism is based on his comments on the 1952 UNESCO statement on “The Race Concept”, the results of an enquiry into “The race question in modern science” (UNESCO 1952). This and earlier reports were largely a response to the extreme antisemitism of the German Nazi regime. The report spends much time on the issue of defining race, emphasising it as a biological concept based on different patterns of frequencies of genetic variation with poorly defined boundaries, and arguing against the notion that races can be characterised by fundamental behavioural differences and so against racist concepts based on such differences. Such racism is characteristically associated with the notion of one race being intrinsically superior to all others.

Fisher expressed the view (UNESCO 1952, p 56) that, since mental abilities were inherited by the same laws as any other differences between people, and because human population groups however defined are likely to differ in the frequencies of genetic polymorphisms affecting behavioural and related traits, one should anticipate the possibility that there would be sufficient differences in the frequencies of such genetic polymorphisms between populations that they would give rise to genetically based, perceived behavioural and other differences between population groups. In his response to the UNESCO document Fisher thus suggests “.. to vary conclusion (2) on page 5, ‘available scientific knowledge provides a firm basis for believing that the groups of mankind differ in their innate capacity for intellectual and emotional development’, seeing that such groups do differ undoubtedly in a very large number of their genes.” (UNESCO 1952, p 56). Here, as elsewhere, he is using the term “gene” for what would now be called a variant of a gene or a genetic polymorphism. The UNESCO document says that Fisher concludes from this that the “practical international problem is that of learning to share the resources of this planet amicably with persons of materially different nature and that this problem is being obscured by entirely well intentioned efforts to minimise the real differences that exist.” (UNESCO 1952, p 27). Fisher expressed a somewhat similar view in a 1954 letter to the geneticist Ruggles Gates (1954-08-27), which ended in the statement “I am sorry that there should be propaganda in favour of miscegenation in North America as I am sure it can do nothing but harm. Is it beyond human endeavour to give and justly administer equal rights to all citizens without fooling ourselves that these are equivalent items?”.

The view that differences in genetically based mental characteristics between races are likely to exist was also expressed in their submissions to the UNESCO document by Hermann Muller (UNESCO 1952, pp 52–55), who was notably left wing in his political views in sharp contrast to Fisher, and the distinguished *Drosophila* geneticist, A.H. Sturtevant (UNESCO 1952, pp 55–56). However, they gave more weight to environmental influences, especially in later years, than did Fisher.

### Was Fisher a racist?

Nearly all of Fisher’s statements were about populations, groups of populations, or the human species as a whole. In addition, Fisher’s discussion of the consequences of race mixture in humans (Fisher 1930a, pp 238–239) dispels any notion that he was a racist in the Nazi and white supremacist sense of believing in the importance of racial purity. In his writings, he did not explicitly mention white Europeans, British people, colonists, slaves, or members of any particular geographical region or group with a particular skin colour, and did not explicitly imply in his comments a superiority of one group over another, which is what many consider to be the essence of racism.

We note, however, that there are also valid concerns with racism in a much broader context and, more generally, with views that lead to discrimination against members of particular ethnic and religious groups. There is no doubt that views that might now be considered racist in this broader sense were widespread in British society when Fisher was addressing eugenic issues, and may have influenced Fisher’s thinking on these issues (Kevles 1985). In addition, it is likely that some of his writings would be viewed as inappropriate if written at the present time, most notably his statements made in the context of the UNESCO enquiry into race, given that he had, as discussed earlier, previously viewed human value in terms of capacity for intellectual development.

Nevertheless, Fisher’s involvement in eugenics and related issues provides no support for the view that he was a racist in the stronger sense of supporting racial discrimination. When he succeeded Karl Pearson as the Galton Professor of Eugenics in University College London, he changed the sub-heading of the Annals of Eugenics from Pearson’s “for the scientific study of racial problems” to “devoted to the genetic study of human populations” (Kevles 1985, p 211).

**Fig. 2:**
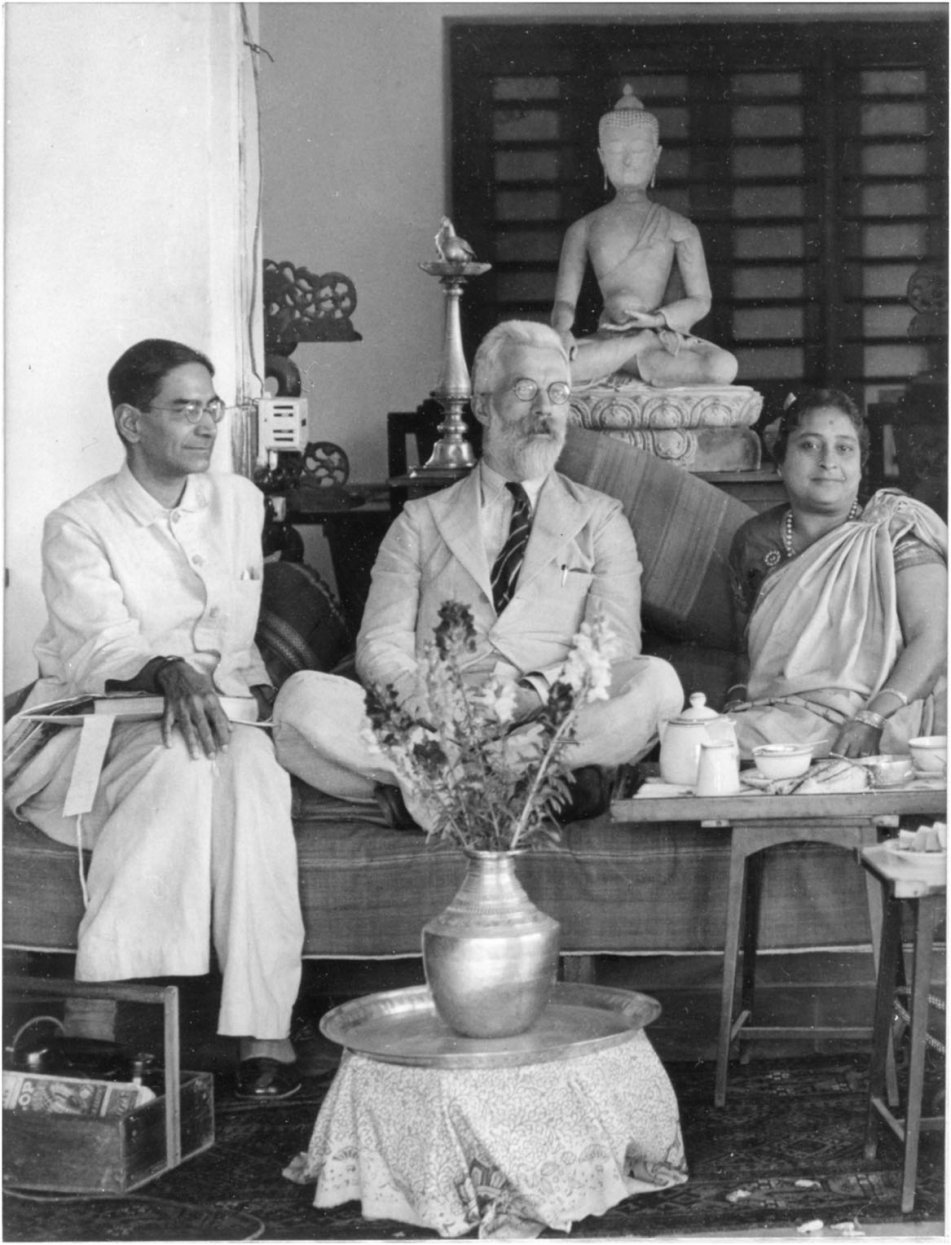
Fisher in the company of Professor Prasanta Chandra Mahalanobis and Mrs. Nirmalkumari Mahalanobis in India in 1940. Courtesy of the P.C. Mahalanobis Memorial Museum and Archives, Indian Statistical Institute, Kolkata, and Rare Books and Manuscripts, University of Adelaide Library.

On a personal level, Fisher had a very close relationship with the pioneer of Indian statistics, P.C. Mahalanobis (Fig. 2), some of whose co-workers came to work with Fisher in London and Cambridge (Rao 1992). He strongly and publicly supported Mahalanobis’ scheme, using the principle of random sampling, for the Indian National Sample survey, including by writing to the Viceroy of India (Rudra 1997). His obituary in *Sankhya* stated that “Through his several visits to the Institute, personal contacts with its workers, scientific contributions to *Sankhya*: the Indian Journal of Statistics, visits to other scientific centres and advisory work in India, he helped in a most significant way in the development of the integrated research, training and project programmes of the Indian Statistical Institute and in its emergence as a higher technological institution of a new type, and also generally in the advancement of statistics in India” (Mahalanobis 1962).

**Fig. 3:**
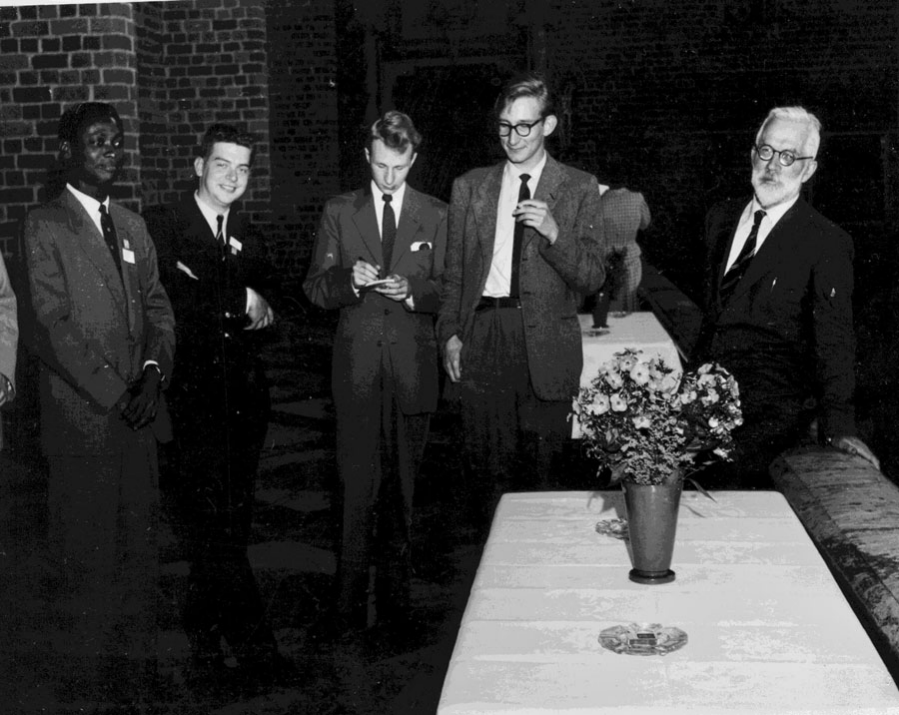
Fisher (far right) in the company of Ben Laing (far left) and Walter Bodmer (second left) at the International Statistics Conference in Stockholm in 1957 (courtesy of W.F. Bodmer).

He appointed Dr. Fabius Gross, who came to England in 1933 as a Jewish refugee from Germany, as an assistant and supported him in his subsequent career (correspondence, Gross 1940-12-05). Among Fisher’s last graduate students in Cambridge were Walter Bodmer, another refugee from Nazi Germany because his father was Jewish, and Ebenezer Laing from Ghana, who became a leading professor of genetics in the University of Ghana (Fig. 3). At Fisher’s funeral, the statistician E.A. Cornish praised Fisher’s “incalculable contribution to the research of literally hundreds of individuals, in the ideas, guidance, and assistance he so generously gave, irrespective of nationality, colour, class, or creed.” (Bennett 1990, pp xvi–viii). Daniel Kevles, in his excellent book *In the Name of Eugenics*, characterises Fisher as an “antiracist conservative” (Kevles 1985, p 170).

## The broader context

The media and social media of the early 21st century have rightly highlighted many social injustices across world society, with concerns raised about historical events and figures stimulated by recent abhorrent events. These heightened sensitivities have led to a reconsideration of the honour given to individuals from preceding times who are felt to have contributed to social injustice in the past, or to have held views that are felt to have promoted social injustice. Recent criticism of R. A. Fisher falls largely into the second category (see e.g. Evans 2020; Tarran 2020) and focusses principally on his involvement in the eugenics movement of the early 20th century, as discussed earlier in this paper. In reconsidering the honours bestowed on individuals from preceding times, it is important to form a balanced view of their impact, and to assess fully the available evidence before drawing conclusions. Hopefully, taking a balanced approach will encourage a rational debate about how such historical events should affect modern culture and thinking and lead to a broader and deeper understanding of relevant scientific research developments. It is especially important to appreciate the context in which research was developed and published, though recognising that current events and thinking might throw a different light on the research and require a new assessment of these historical events.

An important contextual starting point is the contrast between the project-focussed research of the late 20th and early 21st centuries, and the much more flexible research environment of the early 20th century. When Fisher was appointed to work at Rothamsted in 1919, he was asked to apply his statistical and genetic thinking across a range of current agricultural science research challenges, but was also strongly encouraged to engage with the wider scientific community (Box 1978, pp 95–96). This encouragement will have led to his involvement with several topics of common interest at the time, many of which will have influenced his development of key statistical and genetic approaches that are still in common use today, and it is possible that such developments would not have occurred without his exposure to the wider scientific community.

Two further questions of context seem relevant in considering these claims about Fisher’s views on eugenics. The first is to ask how Fisher’s views were regarded by society, and in particular, the intellectual circles of his society. In writing about studies of the history of science, Richard Lewontin (Olby, Lewontin and Kevles 1986) expresses the worry that “by concentrating on the individual creators of ideas or fashions, one may easily fail to ask what social circumstances engendered the problematic in the first place; why they took hold and influenced others, when equally plausible explanations did not; and whether the ideas are part of a larger scientific and social process.” For example, there seems little evidence that Fisher’s advocacy of voluntary sterilisation of some members of society in order to enhance the gene pool was something that generated widespread criticism per se at the time. Indeed, several prominent liberal and left-wing figures, including John Maynard Keynes and Julian Huxley, were members of the Eugenics Society at this time. Also, as outlined earlier, voluntary sterilisation was then widely discussed in scientific circles and investigated by the British Government. Given this, the attempt to evaluate the possible effect of such a programme was regarded as scientifically valuable. As Fisher wrote in 1935, in this regard, “the aim” of our work “is to supply a solidly established body of fact which may be of service to the statesman in framing our laws..¨” because “the task of applying science’s general truths to the needs of a particular nation at a particular period is one for the legislator” (Fisher 1935c).

However, his personal advocacy of a position, derived from information available and assuming goals accepted at the time, may seem problematic and is certainly not now widely acceptable. That leads to the second question of whether it is reasonable to suppose that Fisher would have maintained his views if he had the information available today at his disposal.

The Fisher Memorial Trust, of which the authors are trustees, exists because of Fisher’s foundational contributions to genetical and statistical research. It honours these and the man who made them. Recent criticism of R. A. Fisher concentrates, as we have extensively discussed, on very limited aspects of his work and focusses attention on some of his views, both in terms of science and advocacy. This is entirely appropriate, but in re-assessing his many contributions to society, it is important to consider all aspects, and to respond in a responsible way—we should not forget any negative aspects, but equally not allow the negatives to completely overshadow the substantial benefits to modern scientific research. To deny honour to an individual because they were not perfect, and more importantly were not perfect as assessed from the perspective of hindsight, must be problematic. As Bryan Stevenson (Stevenson 2014) said “Each of us is more than the worst thing we’ve ever done.”

In one of Fisher’s last papers celebrating the centenary of Darwin’s *“The Origin of Species”* and commenting on the early Mendelian geneticists’ refusal to accept the evidence for evolution by natural selection he said, “More attention to the History of Science is needed, as much by scientists as by historians, and especially by biologists, and this should mean a deliberate attempt to understand the thoughts of the great masters of the past, to see in what circumstances or intellectual *milieu* their ideas were formed, where they took the wrong turning track or stopped short of the right” (Fisher 1959). Here, then, there is a lesson for us. Rather than dishonouring Fisher for his eugenic ideas, which we believe do not outweigh his enormous contributions to science and through that to humanity, however much we might not now agree with them, it is surely more important to learn from the history of the development of ideas on race and eugenics, including Fisher’s own scientific work in this area, how we might be more effective in attacking the still widely prevalent racial biases in our society.
